# Tissue Damage, Temperature, and pH Induced by Different Electrode Arrays on Potato Pieces (*Solanum tuberosum* L.)

**DOI:** 10.3389/fonc.2018.00101

**Published:** 2018-04-19

**Authors:** Maraelys Morales González, Claudia Hernández Aguilar, Flavio Arturo Domínguez Pacheco, Luis Enrique Bergues Cabrales, Juan Bory Reyes, Juan José Godina Nava, Paulo Eduardo Ambrosio, Dany Sanchez Domiguez, Victoriano Gustavo Sierra González, Ana Elisa Bergues Pupo, Héctor Manuel Camué Ciria, Elizabeth Issac Alemán, Francisco Monier García, Clara Berenguer Rivas, Evelyn Chacón Reina

**Affiliations:** ^1^Departamento de Farmacia, Facultad de Ciencias Naturales, Universidad de Oriente, Santiago de Cuba, Cuba; ^2^Escuela Superior de Ingeniería Mecánica y Eléctrica (ESIME)-Zacatenco, Instituto Politecnico Nacional, Ciudad de México, México; ^3^Centro Nacional de Electromagnetismo Aplicado (CNEA), Dirección de Ciencia e Innovación Tecnológica, Universidad de Oriente, Santiago de Cuba, Cuba; ^4^Programa de Pós-Graduação em Modelagem Computacional, Departamento de Ciências Exatas e Tecnológicas, Universidade Estadual de Santa Cruz, Ilhéus, Brazil; ^5^Departamento de Física, Centro de Investigaciones Avanzadas del Instituto Politécnico Nacional (CINVESTAV-IPN), México City, Mexico; ^6^Grupo de las Industrias Biotecnológica y Farmacéuticas (BioCubaFarma), Havana, Cuba; ^7^Department Theory and Bio-Systems, Max Planck Institute of Colloids and Interfaces, Potsdam, Germany; ^8^Departamento de Telecomunicaciones, Facultad de Ingeniería Eléctrica, Universidad de Oriente, Santiago de Cuba, Cuba; ^9^Infotur, Holguín, Cuba

**Keywords:** potato (*Solanum tuberosum* L.), electrode array, electrochemical treatment, temperature, pH, sodium hypochlorite

## Abstract

One of the most challenging problems of electrochemical therapy is the design and selection of suitable electrode array for cancer. The aim is to determine how two-dimensional spatial patterns of tissue damage, temperature, and pH induced in pieces of potato (*Solanum tuberosum* L., var. Mondial) depend on electrode array with circular, elliptical, parabolic, and hyperbolic shape. The results show the similarity between the shapes of spatial patterns of tissue damage and electric field intensity, which, like temperature and pH take the same shape of electrode array. The adequate selection of suitable electrodes array requires an integrated analysis that involves, in a unified way, relevant information about the electrochemical process, which is essential to perform more efficiently way the therapeutic planning and the personalized therapy for patients with a cancerous tumor.

## Introduction

Electrochemical therapy (EChT) (1, 2) and electrochemotherapy (ECT) (3) have been targeted for cancer treatment. The EChT involves the application of a very low intensity direct current in the tumor, through electrodes fully inserted in it. The ECT is electroporation, which involves the application of short electrical pulses of high intensity combined with chemotherapy to significantly increase the entry of drugs into tumor cells by electro-permeabilization of their membranes.

Under the framework of the EChT and ECT, efforts have been mainly addressed to the proposal of suitable and efficient electrode arrays for cancer treatment. The electrode array design comprises important technical characteristics such as number, polarity, spacing, form of insertion, type (straight or flexible), and more importantly the electrode geometry. Nevertheless, it has not yet been established an optimal electrode array that allows obtaining the maximum effectiveness administrating EChT with minimal damage to the patient under treatment. Notwithstanding, two-dimensional (2D) (4, 5) and three-dimensional (3D) (6, 7) models have been proposed for their analysis. Such models help to determine how the electric potential, the electric field intensity, the electric current density, and/or the temperature, generated by several different types of electrodes array are distributed in the tumor and the surrounding healthy tissue. These electrode arrays can be interchangeably used in EChT, ECT, and hyperthermia. However, this research focuses mainly on EChT.

Pupo et al. (5) show theoretically how spatial distributions of the electric potential and electric field intensity depend on the shape of the electrode array (circular, elliptical, parabolic, and hyperbolic) for a homogeneous, isotropic, and linear biological medium. In addition, they suggest the hypothesis that the spatial pattern of the tissue damage depends on the shape of the electrode array. Such hypothesis is experimentally confirmed by González et al. (8) in three cross-sections of each untreated or EChT-treated 3D potato piece (*Solanum tuberosum* L.) for electrode arrays with circular, elliptical, parabolic, and hyperbolic shapes, and a collinear electrode arrangement. Additionally, this hypothesis has also been corroborated by other studies for other electrode configurations (9, 10).

Results from González et al. (8) suggest that spatial patterns of tissue damage are similar for the different cross-sections of an EChT-treated 3D potato piece, when the straight electrodes are inserted along it. This finding complies with the fact that the spatial patterns of electric field are similar for the different planes of a 3D biological tissue piece perturbed with EChT (6) or ECT (11, 12), when the straight electrodes are also inserted along it. This may suggest, in a first approximation, that 2D tissue pieces may be used to evaluate other effects induced by any electrode array in them, such as temperature and pH.

Cuban experience suggests that the adequate selection of an electrode array should be conducted by means of an integrated analysis of the spatial profiles of tissue damage, electric potential, electric field, temperature, and pH generated by any electrode array geometry. This issue has not been addressed in literature yet. This integrated knowledge may be relevant for therapeutic planning and personalized EChT/ECT; however, it becomes cumbersome when the biological medium is a solid tumor due to its high complexity. Consequently, the use of less complex biological models is necessary. Such models are required to share characteristics which are similar to those of the tumors, like the potato (*S. tuberosum* L.) does, taking into account the replacement, one 3R (13).

Although the potato and solid tumor are completely different biological systems, these two biological systems exhibit some similar features, as anisotropy, heterogeneity, generally ellipsoidal geometry, irregular edges, and pH ranges (6–7.2), water (60–80%), and electrical conductivity (0.01–0.03 S/m), depending on potato variety (9, 14, 15). These findings have also been previously reported in cancer, depending on its histological variety (16–19). In principle, these similarities between potato and tumor suggest that the potato may be used as a biological model to evaluate the alterations that induce any electrode array in a biological tissue, as in Ref. (8–10). Therefore, the aim is to determine how spatial profiles of tissue damage, temperature, and pH induced in a 2D piece of potato (*S. tuberosum* L., var. Mondial) depend on electrode array with circular, elliptical, parabolic, and hyperbolic shape.

## Materials and Methods

The research is conducted at the Escuela Superior de Ingeniería Mecánica y Eléctrica [ESIME-Zacatenco, Instituto Politécnico Nacional (Ciudad de México, México)]. The experiment is carried out from 8:00 to 10:00 a.m. under controlled conditions of temperature (23 ± 1°C) and relative humidity (65 ± 2%). These two parameters are measured with the instrument Temperature and Humidity Station With Atomic Time (Model: RMR203HG, Resolution 0.1°C, humidity range 25–95%, and humidity resolution 1%, Oregon Scientific, USA).

Three replicates (*N* = 3) of the experiment for each type of electrode array are made in order to confirm the reproducibility of results.

### Biological Model

The potato tuber (*S. tuberosum* L., var. Mondial), which is widely used for research in Mexico (14), is selected as a biological model, and it has been supplied by the Mexican Institute of Genetics for Seed Quality Control. Potatoes are fresh and free of bacteria, viruses, and fungi, according to the phytosanitary certificate. This potato variety is characterized by the following: yellowish-brown color pulp, pH 6.1, and 75% water content (20).

The tuber peel is removed so that its edge is irregular, as observed in tumors (1). Then, potato is transversely cut into pieces of 5 cm × 4.5 cm × 0.2 cm with a potato slicer (Dicer express, China). The size of each potato piece is big enough to determine how the tissue damage, temperature, and pH are 2D distributed around and between the electrodes, as well as in regions away from them, for each electrode array shape. This allows confirming to confirm the validity of 2D theoretical models (4, 5) and corroborates the different experimental findings reported in tumors (1, 16) and potatoes (9, 10).

### Shapes of Electrode Array

**Figure 1:**
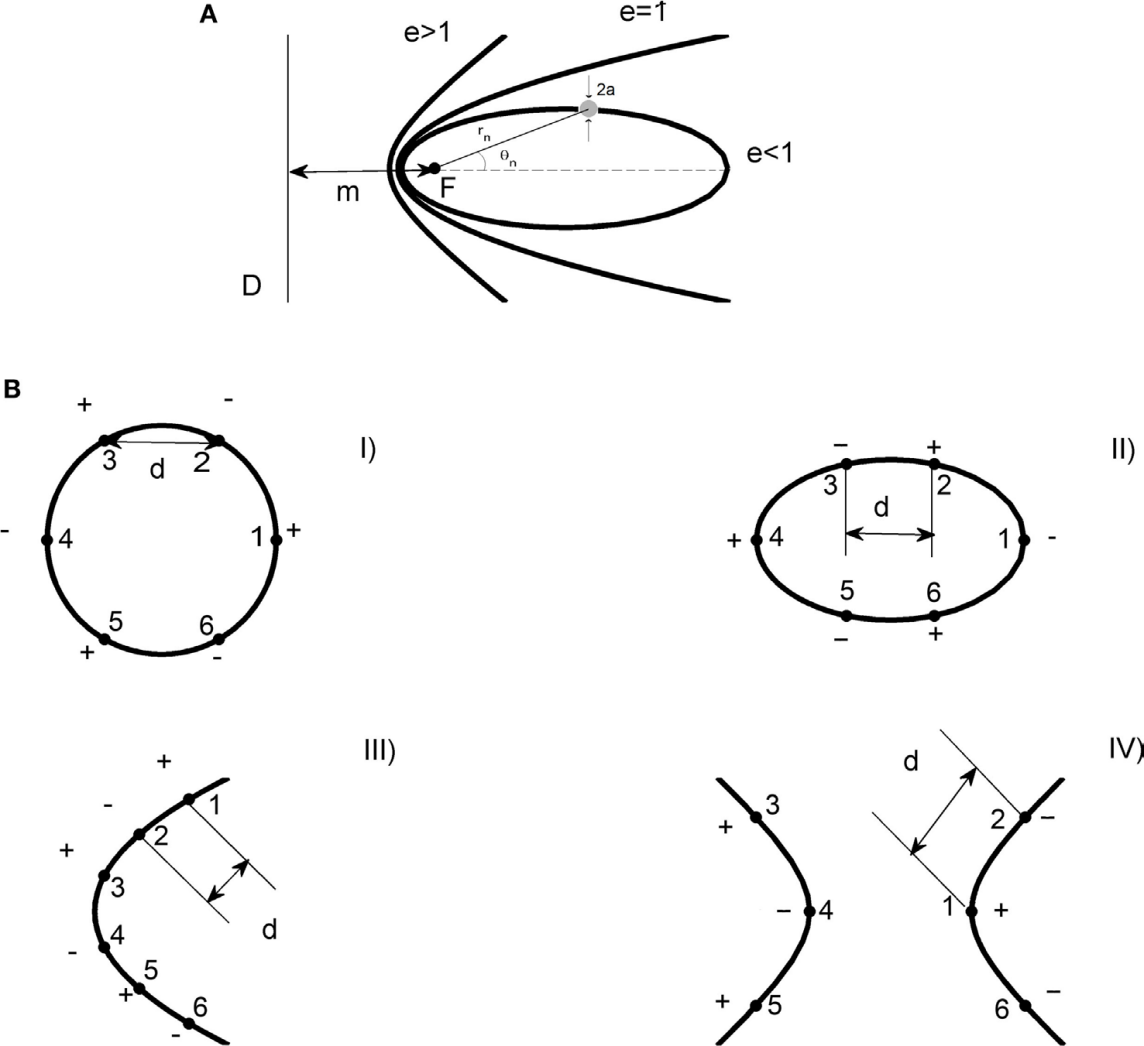
Shapes of electrode arrays. **(A)** Conic sections: ellipse (*e* < 1), parabola (*e* = 1), and hyperbole (*e* > 1). *F* is the focus of the conic section and *a* the radius of the electrode. *D* represents the directrix line and *m* the distance between *F* and *D*. The parameter *d* symbolizes the distance between two consecutive electrodes. *r_n_* and θ*_n_* are the distance and angle of the *n*-th electrode, respectively. **(B)** Electrodes array with circular (Configuration I), elliptical (Configuration II), satellite (Configuration III), and hyperbolic (Configuration IV) shape. The numbering and polarity of each electrode is represented for the four shapes of electrode configurations (6).

The electrode array shape is defined by the unifying principle for the conic sections (5, 8). The circle (Configuration I), the ellipse (Configuration II), the parabola (Configuration III), and hyperbola (Configuration IV) are the conic sections used in this research (Figure 1). The generatrix (*m*) and eccentricity (*e*) of each conic section are shown schematically in Figure 1A. Table 1 shows geometrical characteristics for each electrode configuration.

**Table 1:** Geometrical characteristic for each electrodes configuration.

Electrode array shape	Eccentricity, *e*	Generatrix, *m* (mm)
Circular (Configuration I)	0.0	0.0
Elliptic (Configuration II)	0.6	7.0
Parabolic (Configuration III)	1.0	7.0
Hyperbolic (Configuration IV)	2.0	7.0

The position of the *n*-th electrode in each shape of electrode array is fixed by its polar coordinates (*r_n_*, θ*_n_*), as shown schematically in Figure 1A. *r_n_* is the distance from a reference point to the *n*-th electrode. θ*_n_* is the angle formed between a reference direction (axis *x*) and the *n*-th electrode. Values of *r_n_* and θ*_n_* are shown in Table 2. Besides, these values are referred to the center of the circle, the ellipse, and the hyperbola. In the case of the parabola, *r_n_* and θ*_n_* are referred to its vertex.

**Table 2:** Polar coordinates (*r*, θ) of the six electrodes for each electrode array shape.

Shapes of electrode arrays	Number and positioning of each electrode											
	1		2		3		4		5		6	
	θ (^o^)	*r* (mm)	θ (^o^)	*r* (mm)	θ (^o^)	*r* (mm)	θ (^o^)	*r* (mm)	θ (^o^)	*r* (mm)	θ (^o^)	*r* (mm)
Circular	0	5.00	60	5.00	120	5.00	180	5.00	240	5.00	300	5.00
Elliptic	0	6.56	60	5.50	120	5.50	180	6.56	240	5.50	300	5.50
Parabolic	60	9.33	65	7.20	75	3.88	285	3.88	295	7.20	300	9.33
Hyperbolic	0	4.67	45	8.08	135	8.08	180	4.67	225	8.08	315	8.08

Figure 1 shows the numbering and polarity of each electrode, and the distance *d* between two consecutive electrodes *i-j* (*i* ≠ *j*) for each shape of electrodes array. The positive and negative electrodes are the anodes and cathodes, respectively. For the circle, *d* = 5 mm for consecutive electrodes 1-2; 2-3; 3-4; 4-5; and 5-6. For the ellipse, *d* = 6.10; 5.50; 6.10; 6.10; and 5.50 mm for the adjacent electrodes 1-2; 2-3; 3-4; 4-5; and 5-6, respectively. For the parabola, *d* = 2.25 mm (for the adjacent electrodes 1-2 and 4-5), 3.45 mm (for the next electrodes 2-3 and 5-6), and 7.50 mm (for successive electrodes 3-4). For the hyperbola, *d* = 5.81 mm for 2-1 consecutive electrodes; 1-6; 3-4; and 4-5 (5, 8). The distance between the electrodes is measured by means of a vernier caliper with clamping screw (Model 530-104 of 0.05 mm of precision, Mitutoyo, Japan).

The electrodes are straight needles of platinum–iridium (Pt–Ir 90/10) 10 cm in length and 0.7 mm in diameter.

### Electrochemical Treatment

Six experimental groups are formed: one negative control group (NCG), a positive control group (PCG), and four treated groups for each shape of electrodes array. In the NCG, neither electrodes are inserted nor direct current is applied in the piece of potato. In the PCG, electrodes are inserted in the piece of potato during 30 min and direct current is not applied on it. In all treated groups, the electrodes are inserted into pieces of potato and direct current is applied on them. In the first treated group, the Configuration I (TG1) is used. In the second treated group, the Configuration II (TG2) is used. In the third treated group, the Configuration III (TG3) is applied. In the fourth treated group, the Configuration IV (TG4) is used. The intensity of direct current (10 mA) and exposure time (30 min) remain constant in GT1, GT2, GT3, and GT4, during the application of EChT. The EChT is applied once for each type of electrodes array.

All experimental groups are under the same experimental conditions. For each shape of electrodes array, the electrodes are inserted into the piece of potato so that their tips are far away from this piece. This ensures that the tissue damage, the temperature, and pH are distributed in 2D, as assumed in Ref. (5).

### Electrical Device

The ONCOCED^®^ B&E-01 is used to apply the direct current. This device is designed and built by specialists from the Centro de Biofísica Médica, Centro Provincial de Electromedicina, and Grupo de Bioelectricidad (formed by researchers of the Centro Nacional de Electromagnetismo Aplicado, Universidad de Oriente, BioCubaFarma and different hospitals), all from Santiago de Cuba, Cuba. This equipment allows monitoring the intensity of the direct current and voltage during application of EChT. The values of these two physical quantities are monitored every 5 min during the application of EChT.

### Compact Infrared Camera FLIR

In this paper, the methodology described by Aguilar et al. (21) is followed to obtain thermal images in the piece of potato by means of a compact infrared camera FLIR (Systems Wilsonville, OR, USA). The characteristics of this camera are as follows: i5 model; 6.8 mm lens; accuracy of ±2%; thermal sensitivity <0.1 at 25°C; 140 × 140 of thermal images resolution and temperature range −20 to 250°C. The analysis of these images is carried out by means of the free software (FLIR Tools). The site of this software is http://support.flir.com/SwDownload/app/RssSWDownload.aspx?ID=120.

The thermal images are obtained every 5 min during 30 min of exposure to EChT. The pictures are stored in the camera memory of thermography for later use. Data matrices are obtained by the FLIR Tools software. These matrices are processed in the free software GNU Octave 4.0 (License 29.5.2015) for 2D representation of temperature distributions in the piece of potato and its environment. The GNU Octave software site is http://gnu.org/software/octave.

### pH Measurement

The pH of the piece of tuber is measured by means of an universal pH indicator paper (range 1–14, Q/GHSC1544-2006, Shanghai SSS REAGENT Co., Ltd., China) 30 min after starting the experiment. A universal pH indicator paper is used because it is a universal method, and its results comply with those obtained with pH-meters in tumors (16). In the NCG, pH is measured randomly in different parts of the potato piece. In the PCG and the four treated groups, the pH is measured around and between the electrodes, as well as on areas away from them.

### Macroscopic Observations

The color of each piece of potato is the macroscopic finding reported in the first 120 min after initiating the experiment in each experimental group. The change of color (visual attribute) of the potato is an indicator of appearance, possible damage, and quality of this tuber (22).

The images of the pieces of potato in all experimental groups are obtained 30 min after initiating the experiment, with a Kodak EasyShare (model M340 digital camera; 4-GB memory card; 10.2 megapixels; Eastman Kodak Company, New York, NY, USA).

## Results

### Tissue Damage

**Figure 2:**
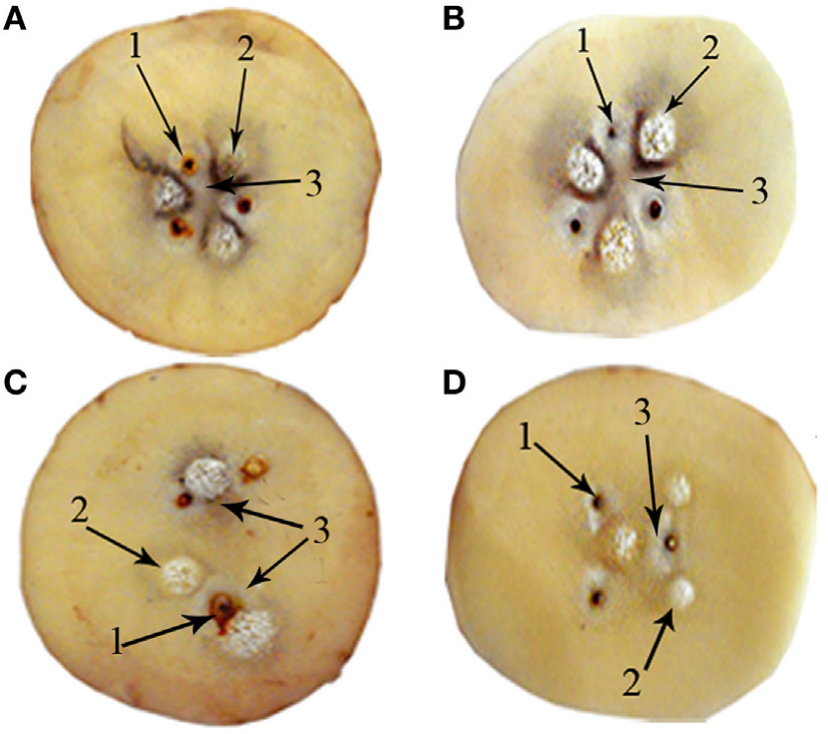
Findings of the potato pieces perturbed with 10 mA during 30 min for **(A)** Configuration I; **(B)** Configuration II; **(C)** Configuration III; and **(D)** Configuration IV. In this figure is shown the necrosis around each electrode (1), bubbling around the cathode (2), and irreversible tissue damage between the electrodes (3).

Figure 2 shows the 2D spatial distribution of tissue damage induced in the pieces of potato (*S. tuberosum* L., var. Mondial) by the action of 10 mA during 30 min for TG1, TG2, TG3, and TG4. This damage is represented by dark coloration that is observed in tuber pieces around and between the electrodes. The form of this spatial distribution is similar to that of the electrode array, as it is evidenced in Figures 2A–D for circular, elliptical, parabolic, and hyperbolic shapes, respectively.

The dark coloration with circular shape around each anode and cathode, and irreversible tissue damage between the electrodes are observed in the potato pieces in each treated group (Figure 2). In addition, the texture of the potato piece around and between electrodes changes during EChT application.

Figure 2 shows that the severity of tissue damage is proportional to the coloration intensity. The greatest intensity and extent of coloration are observed for circular and elliptical electrode arrays. Additionally, the areas of the potato piece away from the electrodes are not affected during the application of EChT.

Even if the results are not displayed, the dark coloration of the piece of potato among electrodes begins to be observed after 10 min of exposure of EChT for TG1, TG2, TG3, and TG4. This darkening extended in space and time begins to be noticeable when the exposure time of EChT increases. This fact is observed during 90 min after treatment.

In the pieces of potato of PCG, tenuous dark areas with circular shape only around each electrode are evidenced. The parts of the pieces of potato away from electrodes are not affected by inserting them. These findings are observed for the four shapes of electrode configurations without the application of EChT. The experimental findings evidenced in PCG and on each of the four treated groups are absent in NCG. Furthermore, changes in texture are not observed in the pieces of potato of NCG and PCG.

A tenuous brown coloration begins to be observed in the potato pieces for all experimental groups after withdrawing its peel. This coloring appears randomly and slowly over time, and differs from that around and between the electrodes when applying EChT (Figure 2).

### Electric Field Intensity

**Figure 3:**
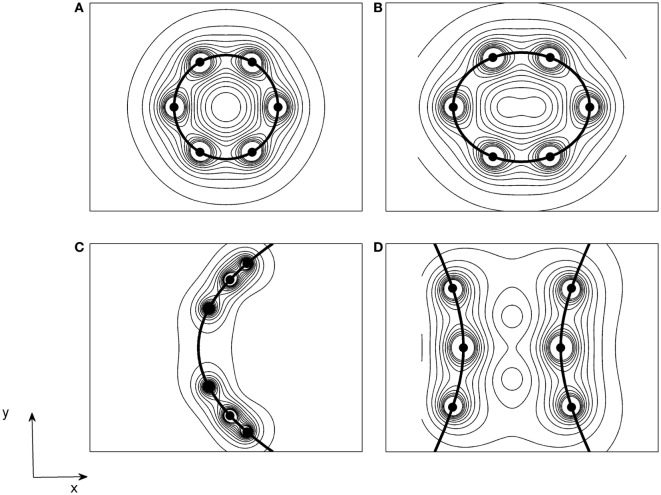
Two-dimensional spatial patterns of the electric field intensity induced in an homogeneous, isotropic, and linear medium for electrode configurations with **(A)** circular; **(B)** elliptical; **(C)** parabolic; and **(D)** hyperbolic shapes, which are defined in Figure 1B [courtesy of Pupo et al. (6)].

Even though the piece of tuber is heterogeneous tissue, the theoretical results of Pupo et al. (5) are also included in this paper to verify if there is a similarity between the profiles of 2D spatial distributions of tissue damage in the potato pieces and the electric field intensity induced by the electrode arrays with circular (Figure 3A), elliptical (Figure 3B), parabolic (Figure 3C), and hyperbolic (Figure 3D) shapes.

The results of Figures 2 and 3 show that there are similarities between the forms of the spatial patterns of tissue damage and the electric field intensity for the four shapes of electrodes arrays. The forms of these spatial patterns take the shape of electrode array. Furthermore, these figures show that the tissue damage and the lines of the electric field intensity have circular shapes around the electrodes.

### Temperature

**Figure 4:**
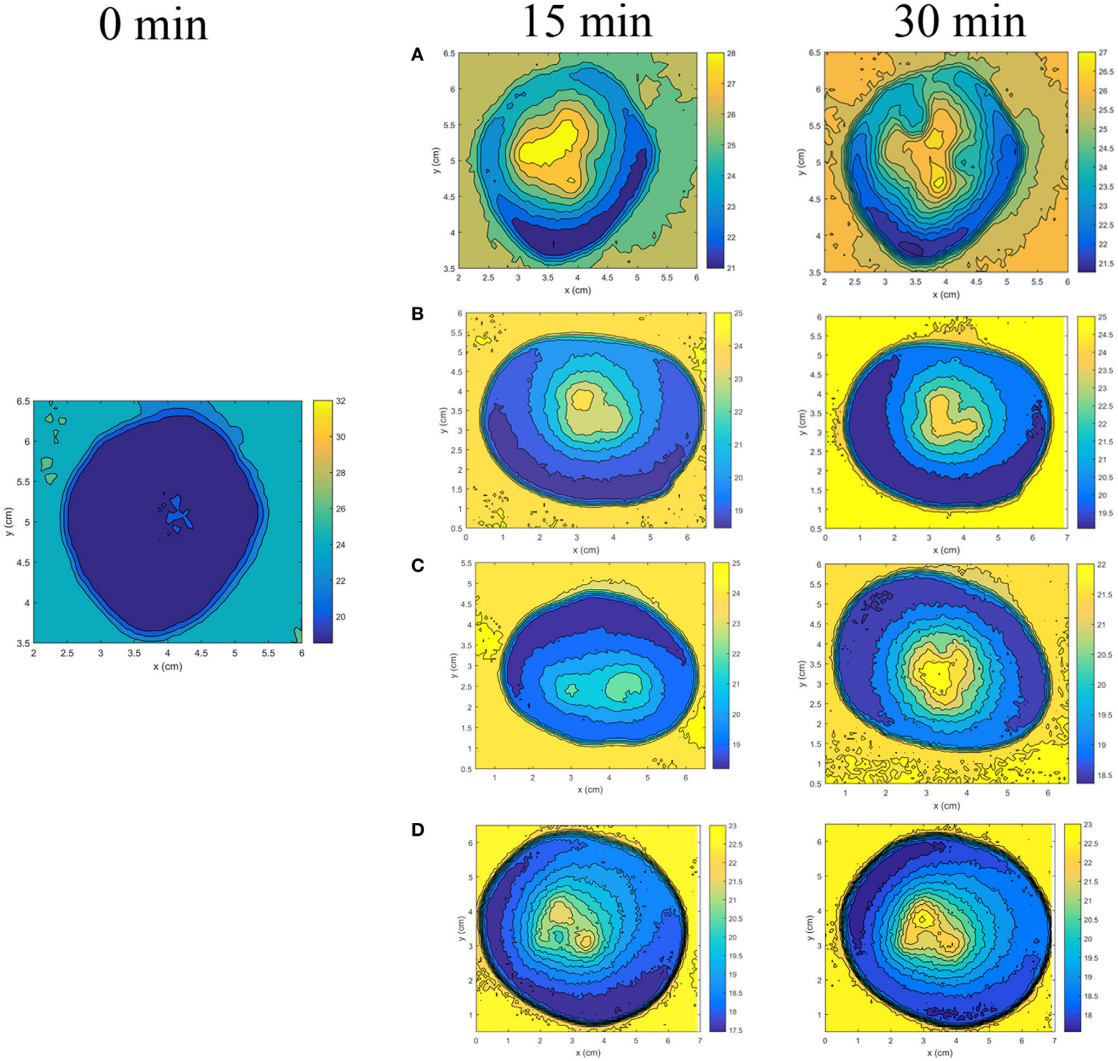
Two-dimensional temperature spatial distribution induced in the potato piece at 0, 15, and 30 min of electrochemical therapy exposure for the electrode arrays with **(A)** circular; **(B)** elliptical; **(C)** parabolic; and **(D)** hyperbolic shapes. The temperature pattern shown at 0 min is similar to that observed in positive control group and negative control group at 15 and 30 min of observation.

Figure 4 shows how the spatial distributions of the temperature change in the pieces of tuber of the experimental groups at 0, 15, and 30 min. Temperature distributions are similar for NCG and PCG. They do not change during the 30 min of observation. That is why, a temperature spatial pattern is only presented for NCG and PCG, which may be also used as a reference for the four treated groups (Figure 3A).

An entirely different scenario is observed in EChT-perturbed potato pieces for electrode configurations of circular (Figure 4A), elliptical (Figure 4B), parabolic (Figure 4C), and hyperbolic (Figure 4D) shapes. These figures show that temperature is distributed radial and not uniformly in the potato pieces around and between the electrodes, being more intense around each electrode. The temperature of potato pieces at 30 min of EChT exposure is higher than values measured at *t* = 0 for each electrodes array shape. Its spatial distribution depends on the electrode array shape and decreases when increasing distance where electrodes (heat sources) are inserted. Likewise, the temperature is not changed for parts of the potato pieces far from electrodes. The electrode circular array concentrates more on the temperature inside the potato model than the other forms of electrodes array.

Moreover, the temperature outside the central region into the potato piece increases during the 30 min of EChT exposure in all treated groups (Figure 4). By contrast, it is not modified during the 30 min of observation for NCG and PCG.

### pH and Other Electrochemical Processes

In all treated groups, the pH around each anode and cathode is acidic (red, pH ≤ 4) and basic (blue, pH ≥ 10) respectively. The pH is acidic (orange, 4 < pH ≤ 6) between the electrodes and slightly acidic (orange-yellow, 6 < pH < 7) in areas away from electrodes.

In NCG and PCG, the pH is slightly acidic in the entire area of the potato pieces.

Other electrochemical processes are also observed, for instance: dehydration and watering are also observed around anode and cathode, respectively; a white color is seen at the vicinity of each anode; bubbling in the vicinity of each cathode and chlorine gas smell is perceived during the application of EChT for TG1, TG2, TG3, and TG4.

A dark color at the tip of each anode is observed after EChT application. After each treatment, the surface of each anode is polished with a very fine sandpaper to eliminate this dark coloration, and then all electrodes are cleaned and sterilized in alcohol 70% prior to use.

## Discussion

### Temperature

The increase of temperature around and between electrodes is explained by heating due to the Joule effect (9, 23). This heating results from the electric power dissipated in this region of the potato piece. Technically, this electric energy depends on the exposure time of EChT, the square of the induced electric field intensity, and the electrical conductivity of the tuber piece. Although electrical conductivity in these potato pieces is not measured in this study, it increases with the temperature, as documented in Ref. (9, 23).

The increase of the electrical power released around and between the electrodes is corroborated in this study because the voltage increases non-linearly during the application of EChT (results not shown). This increase of the voltages may be explained from non-linear variations of the electrical resistance of the potato piece due to physical–chemical processes induced in it.

On the other hand, anisotropy/heterogeneity of the potato piece may explain the non-linear behavior of the temperature profile. In solid tumors, this non-linear behavior of the temperature profile induced by EChT can be explained from their anisotropy/heterogeneity and vascularization degree. The tumor angiogenesis is a correlate of aggressiveness and quick growth of tumor (24). Solid tumors are vascularized, whereas the potato is not vascularized. Xin et al. (2) report different global effectiveness percentages for several solid tumor types (highly and poorly vascularized). Therefore, the mechanism that damages potato piece by EChT cannot be extrapolated to tumors because both biological tissues are very different. Moreover, the lack of vascularity process in the potato constitutes the main limitations of this study.

The increase of temperature during the application of EChT may explain the reversible skin erythema observed in the entire area treated in the patient (25). Additionally, this erythema may be due to tissue dehydration.

### pH and Other Electrochemical Processes

Bubbling around the cathode can be explained from the formation of hydrogen gas by means of the water decomposition at this electrode (2H_2_O + 2e^−^ → H_2_ + 2OH^−^) (16, 26). Bubbling is also seen around the cathode in EChT-treated tumors (10, 16, 25) (see Appendix). This finding may suggest that bubbling, *a priori*, identifies the negative electrode polarity for any electrode array in a biological tissue given.

Dehydration around anode and watering around cathode have been explained by water movement from anode to cathode, as reported also in EChT-treated tumors (16, 27). The smell of chlorine gas perceived during the EChT application can be explained by chlorine ions formation at the anode (2Cl^−^ + 2e^−^ → Cl_2_) (16, 26). Cury et al. (27) explain the white zone around each anode as a result Cl_2_ gas formation.

Measurement results of pH in EChT-treated potato piece areas around and between the electrodes, and away from them comply with those reported in EChT-treated tumors (16). Acidity around the anode is explained by formation of hydrochloric acid (HCl: H^+^ + Cl^−^ → HCl) (16) or hypochlorous acid (HClO: Cl_2_(aq) +H_2_O → HClO + H^+^ + Cl^−^) (28). Basicity around the cathode is due the formation of sodium hydroxide ($\left(\text{NaOH: }{\text{N}}_{\text{a}}^{+}+{\text{OH}}^{-}\to \text{NaOH}\right)$ (16). ${\text{N}}_{\text{a}}^{+}$ and Cl^−^ ions are a result of the electrolytic decomposition of sodium chloride $\left(\text{NaCl: NaCl}\to {\text{N}}_{\text{a}}^{+}+{\text{Cl}}^{-}\right)$ (16). H^+^ ion results from the electrolytic decomposition of the water at the anode (2H_2_O → O_2_ + 4H^+^ + 4e^−^), whereas OH^−^ ion results from the water decomposition (16, 26–28). Griffin et al. (26) propose the water decomposition from ${\text{3H}}_{2}\text{O}-2{\text{e}}^{-}\to 2{\text{H}}_{3}{\text{O}}^{+}+\frac{1}{2}{\text{O}}_{2}$. The oxygen gas (O_2_) released at the anode is explained by the water electrolytic decomposition at the anode. In addition, other electrochemical phenomena are induced in the tumor (16) and in the interface electrode/tissue (29, 30). Cabrales et al. (31) suggest the formation of oxidative stress from the anion superoxide, $•{\text{O}}_{2}^{-}$, at the anode $\left({\text{O}}_{2}+{\text{e}}^{-}\to •{\text{O}}_{2}^{-}\right)$. Besides, this anion has also been suggested by Miklavčič et al. (30).

Dark color at the tip of each anode may be explained by the electrode corrosion due to the acidic environment around it (6). Metal corrosion may be linked to the electrode surface roughness, indicating the existence of wear on the anode. Surface roughness in four types of needle electrodes (Pt/titanium, Pt/tungsten, Pt/brass, and Pt/stainless steel) is observed by means of reflected light micrographs (29). This corrosion processes bring about an increase of both polarization and electrical resistance of the electrode. If this becomes noticeable, then direct current intensity drops. Therefore, the anode surface is sanded and cleaned after each treatment.

In contrast with Ref. (16), the authors of this paper consider that NaOH is formed from the sodium metal, Na(s), which appears because ${\text{N}}_{\text{a}}^{+}$ ion acquires an electron (*e*^–^) $\left({\text{N}}_{\text{a}}^{+}+{\text{e}}^{-}\to \text{Na}(\text{s})\right)$. Na(s) then immediately reacts with water to produce NaOH and H_2_ gas by means of the chemical reaction: 2Na(s) + 2H_2_O → 2NaOH +H_2_ + Δ*Q*(Δ*Q* > 0). Δ*Q* > 0 means that the reaction is exothermic. This fact may explain the heating of cathode in this experiment and in tumors (25). Δ*Q* depends on the direct current intensity and the exposure time, thereby leading to mass transport (ionic diffusion) due to temperature transference. Additionally, HCl and HClO can be explained from chlorine hydrolysis: Cl_2_ + H_2_O → HClO + HCl.

On the other hand, chlorine odor is perceived because Cl_2_ gas escapes through tissue-air interface. The white zone around each anode may be described by the formation of the sodium hypochlorite (NaClO), by means of the following reaction: NaCl + H_2_O + energy → NaClO + H_2_. This energy is supplied by the direct current that flows through the tumor. The formation of the NaClO is more stable than that HClO, and its odor is similar to the Cl_2_ gas. It may be explained the formation of HClO and NaOH $\left(\text{NaClO}+{\text{H}}_{2}\text{O}↔\text{NaOH}+\text{HClO}↔{\text{N}}_{\text{a}}^{+}+{\text{OH}}^{-}+{\text{H}}^{+}+{\text{ClO}}^{-}\right)$, Cl_2_ gas (NaClO + 2HCl → Cl_2_ + NaCl + H_2_O), and oxygen nascent O (NaClO → NaCl + O) from NaClO. Furthermore, the O_2_ gas may be explained by NaClO and hydrogen peroxide (H_2_O_2_) by means of NaClO + H_2_O_2_ → O_2_ + NaCl + H_2_O (24). H_2_O_2_ is formed at the anode when a direct current is applied to the tumor (O_2_ + H_2_O + 2e^−^ → H_2_O_2_ + 2OH^−^) (30).

NaClO and H_2_O_2_ are strong oxidizing, antiseptic, germicide, and antibacterial agents and have high ability to dissolve tissues. NaClO changes mechanical properties and increases the roughness of a tissue (32), as observed in potato pieces and in Ref. (8). Additionally, NaClO may explain the potato tissue dissolution at the anode (hole); findings observed in other studies in potato (10) and tumors (25). On the other hand, H_2_O_2_, HClO, and others oxidants (reactive oxygen species, such as $•{\text{O}}_{2}^{-}$) may explain the apoptosis and necrosis of cancer cells during and after EChT application (1, 33). Apoptosis in cancer cells is initiated by reactive oxygen species generation in response to EChT (1, 34). These issues may also be relevant to explain the tissue damage in potato pieces.

### Temperature + pH

Electrochemical processes and temperature induced in the piece of potato of the four treated groups can explain the dark coloration around and between electrodes during and after 90 min of EChT application. This result complies with Reyes and Cisneros-Zevallos (22), who report that pH and temperature are parameters that affect the dynamic stability of the chemical reactions and color of potato. Furthermore, the increase in temperature leads to denaturation of protein and other biological structures (7).

Temperature, pH, and oxygen concentration, among other factors, may enhance and accelerate the oxidative process around and between the electrodes. This hypothesis may be argued since these factors influence the activity of the polyphenol oxidase enzyme, which is present in the oxidative processes (15, 35–37).

The irreversible and extensive damage in space and time of the zones of the tuber piece between the anodes and cathodes can be explained by the decrease of concentrations of phenolic compounds as a result of the combination of electrical field intensity, temperature, and time, as Pereira et al. (37) suggest. This space–time extension of this dark coloration has also been confirmed in potato (*S. tuberosum* sp.) (9) and in solid tumors [*in vitro* (38), preclinical (16, 27, 39), and clinical (2, 25) studies].

Even though the temperature effect is considered in this paper, results shown in Figure 2 suggest that electrochemical processes prevail over thermic ones, in accordance with Ref. (27). Electrochemical processes and their effects in the tumor appear on a smaller time scale than that for the appearance of the thermic processes. In other words, electrochemical processes occur faster than thermic processes. This confirms that predominate mechanism of EChT is the induction of toxic products from electrochemical reactions, according to Ref. (2, 16, 26, 27, 29, 38, 39).

The increase of the temperature (40) and the drastic change of pH (32) potentiate the tissue dissolution ability of the NaClO.

### Tissue Damage

The circular necrosis induced by EChT around each electrode has been demonstrated in potato stimulated by ECT (9, 10) and in tumors perturbed with EChT (2, 38). Such tissue damage around and between the electrodes may be explained by the modification of their viscoelastic properties caused by the electromechanical stress tensor induced in the areas, in accord with Pereira et al. (41).

In the PCG, the dark coloration observed around the electrodes is explained by the mechanical disruption that they produce. Moreover, the color changes occur randomly and slowly over time. These are observed in pieces of potato of NCG and in areas away from electrodes (PCG, TG1, TG2, TG3, and TG4) are due to the natural physiological processes of the oxidation and aging of these pieces (15, 36). The natural physiological processes of oxidation can be explained by the release of intracellular enzymes (e.g., polyphenol oxidase) (9, 33, 42). Aging causes about physiological and biochemical changes in the potato tissue (43) and has been explained by the decrease in membrane integrity due to peroxidative damage of membrane lipids by increasing concentrations of ethanol and malonaldehyde (44) among others (45).

### Electric Field + Tissue Damage

The fact that spatial patterns of tissue damage and electric field intensity adopt the electrode array geometry permits to select *a priori* an electrode array depending on the tumor shape and confirms that the tissue damage depends on the electric field intensity induced in it. This similarity has been confirmed by other electrode configurations in potato model (9, 10) and malignant tumors (31, 38, 46, 47). It is expected that these profiles shall become even more similar as the thickness of the potato piece decreases (i.e., thicknesses microns or less). Although the potato and the tumor are completely different biological systems, the similarity between profiles of spatial patterns of tissue damage in the potato showed in this experimental setup (for six electrodes) and those shown in Ref. (46) for four electrodes, when an electrode circular array is used.

Taking into account that the last observation concerns the dark color that appears around and between the electrodes in EChT-treated potato pieces, it may also be interpreted as electrochemical ablation zone (46). In medicine, ablation term refers to a complete destruction of an organ or tissue, by surgery or by physical agents or chemical compounds.

It has been verified that these ablation zones are caused by thermal coagulation (46), similar to the coagulative necrosis observed in EChT-treated tumors (22, 31). This sort of necrosis may also explain, in part, the texture change caused by the application of EChT-perturbing potato pieces for four shapes of electrode arrays.

Otherwise, the similarity between the forms of spatial patterns of tissue damage and electric field intensity for the four shapes of electrode arrays confirm the validity of the 2D model reported by Pupo et al. (5) and Soba et al. (48). Recently, Soba et al. (48) demonstrated theoretically that the electric potential, electric field intensity, temperature, pH, and tissue damage spatial distributions adopt the electrode geometry for these four-electrode arrays and the tissue damage increases over time, according to our experimental findings. In addition, all findings in 2D potato pieces coincide with those observed in 3D potato pieces (8). These two aspects may suggest that 2D models [that consider isotropic, homogeneous, and linear media (5, 6)] may be used, in a first approach, to determine how the electric field intensity distribution depends on the electrode array shape in heterogeneous media, like the potato piece. Moreover, Soba et al. (48) propose an explanation of how the temperature, pH, and combination of temperature and pH can damage to the tumor tissue.

### Future Perspective

Further study is required to quantify how different the approximate results of Pupo et al. (6) are from those obtained when a “realistic model” that incorporates the geometry, anisotropy, heterogeneity, and electrical properties of the medium is used (49). This will shed light on determining if these differences have any significant implications from a therapeutic point of view.

Results of this study set the basis to go deeper into the electrochemical mechanism of the formation of NaClO (patent in process) and the role of the chlorine species in the tumor cell apoptosis, as documented by Holandino et al. (1). This study and the results of Ref. (5, 6, 8, 24, 48) suggest that an integrated analysis of these spatial distributions with biological tissue characteristics (electrical and biological properties) is necessary in order to select adequately an efficacious electrodes array, taking into account electrode geometries similar to those reported in Ref. (8, 49). Additionally, this study strongly recommends to perform a different integrated analysis based on Dynamical Complex System tools of all these physical and chemical quantities as a whole by studying their changing behavior taking into account the electrodes array shape.

In conclusion, the similarity found between the profiles of the spatial patterns on the tissue damage into the pieces of potato (*S. tuberosum L*., *var. Mondial*) and the electric field intensity obtained by themselves; the electrodes array shapes used to confirm the validity of the 2D model reported by Pupo et al. (5). This study is an essential requirement to select optimal parameters, which are of vital importance to perform successfully both the therapeutic planning and personalized therapy for patients with cancerous tumors.

## Availability of Data and Materials

Data sharing not applicable to this article as no datasets are generated or analyzed during the current study, except the temperature data.

## Ethics Statement

This study is approved at the Escuela Superior de Ingeniería Mecánica y Eléctrica (ESIME)-Zacatenco. Instituto Politécnico Nacional México, Ciudad de México, México.

## Author Contributions

Study concepts: MG, CA, FP, LC, JR, VG, and AP. Study design: MG, CA, FP, LC, JR, JN, PA, DD, VG, and AP. Data acquisition: MG, CA, FP, LC, and JR. Quality control of data and algorithms: MG and LC. Data analysis and interpretation: MG, CA, FP, LC, JR, JN, PA, DD, VG, AP, HC, EA, FG, CR, and ER. Statistical analysis: MG and LC. Manuscript preparation: MG, CA, FP, LC, JR, JN, PA, DD, VG, AP, HC, EA, FG, CR, and ER. Manuscript editing: MG, CA, LC, and JN. Manuscript review: MG, CA, FP, LC, JR, JN, PA, DD, VG, AP, HC, EA, FG, CR, and ER.

## Conflict of Interest Statement

The authors declare that the research was conducted in the absence of any commercial or financial relationships that could be construed as a potential conflict of interest.

## Figures and Tables

**Figure A1:**
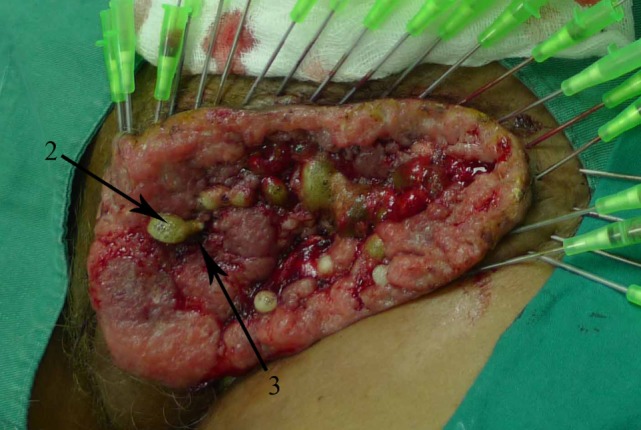
Patient with exophytic vulva tumor treated with 10 mA for 60 min. Bubbling around the cathode (2) and irreversible tissue damage between the electrodes (3). Courtesy of Dr. Luis Bergues Cabrales (unpublished results).

## Appendix

Bubbling at the cathode in potato pieces perturbed with EChT are also observed at this electrode when tumors growing in mice and patients are treated with this therapy. Although research in patients is not carried out in this paper, this experimental finding in a patient (gender: female, age: 72 years old, skin color: black) with exophytic vulva tumor is evidenced in this Appendix. This patient is treated with 10 mA for 60 min, as shown in Figure A1 (unpublished results of the current Pilot study, courtesy of Dr. Luis Bergues, Head of the Bioelectricity group). This patient fails to all oncoespecific methods (surgery, chemotherapy, and radiotherapy) and bleeds frequently, requiring blood transfusion every day. After EChT application, tumor reduces its volume in a 45% and it is evidenced the strong hemostatic effect of the cathode immediately after treatment. As that medical research involves human subjects, it is conducted according to the World Medical Association Declaration of Helsinki. Additionally, it has been approved by the Ethical Committee and Scientific Board of the Oncologic hospital of Santiago de Cuba, Cuba. This Pilot study follows good clinical and medical practices established by the Ministry of Public Health of the Cuba Republic.
